# Reduced Pineal Gland Volume in Oncology Patients: Association with Chemotherapy Duration

**DOI:** 10.3390/medicina61111923

**Published:** 2025-10-27

**Authors:** Milica Šarošković, Miloš Vuković, Jelena Vasić, Igor Nosek, Duško Kozić

**Affiliations:** 1 Faculty of Medicine, University in Novi Sad, 21000 Novi Sad, Serbia; 015033@mf.uns.ac.rs (M.Š.); 015167@mf.uns.ac.rs (J.V.); igor.nosek@mf.uns.ac.rs (I.N.); dusko.kozic@mf.uns.ac.rs (D.K.); 2 Department for Radiology Diagnostics, Oncology Institute of Vojvodina, 21208 Sremska Kamenica, Serbia

**Keywords:** pineal gland, chemotherapy, volume, malignancy, melatonin, neuroendocrine regulation

## Abstract

*Background and Objectives*: The pineal gland is a neuroendocrine structure whose function can be disrupted in patients with malignancies. This study examines the differences in pineal gland volume between oncology patients and healthy controls, as well as the relationship between volume and the duration of chemotherapy. *Materials and Methods*: A retrospective study included 400 participants, divided into two groups: 200 oncology patients and 200 healthy controls. The pineal gland volume was measured using MRI scans, utilizing T1-sagittal, T2-coronal/axial sections, and post-contrast 3D T1W MPRAGE tomograms. The volume was calculated based on the ellipse approximation formula: V = (L × H × W)/2. The study analyzed the relationships between pineal gland volume and factors such as age, sex, primary tumor origin, and the duration of chemotherapy. *Results*: The pineal gland volume was significantly smaller in oncology patients in comparison with the healthy controls (*p* < 0.001). The average volume in oncology patients was 32.41 ± 16.79 mm^3^, whereas in healthy controls, it was 59.26 ± 29.99 mm^3^. A significantly smaller pineal gland volume was observed in patients with malignancies, regardless of sex, with no notable differences between groups. Age also did not significantly influence gland volume (*p* > 0.05). The primary tumor site did not significantly influence gland volume (*p* > 0.05). A moderate positive correlation was observed between the duration of chemotherapy and pineal gland volume (ρ = 0.322; *p* = 0.007). *Conclusions*: The pineal gland showed reduced volume in oncology patients compared to controls. The observed positive correlation with chemotherapy duration should be interpreted cautiously and may reflect survivorship bias rather than direct treatment effects.

## 1. Introduction

The pineal gland, a neuroendocrine structure in the epithalamus, regulates circadian rhythms, seasonal processes, and immune functions. It primarily synthesizes melatonin, a hormone that peaks at night and synchronizes biological clocks with external light, maintaining homeostasis in metabolism, immunity, and sleep [1,2].

Beyond sleep regulation, melatonin has antioxidant and immunomodulatory properties, protecting cells from oxidative stress, a common consequence of malignancies. It also exhibits antiproliferative effects that inhibit tumor growth [3,4]. However, malignancies significantly alter pineal gland function and structure, complicating disease progression and therapy response.

Histopathological analyses reveal degenerative pineal changes in oncology patients, including cystic cavities, gliosis, and increased calcifications, correlating with disease stage, tumor type, and treatment intensity [5,6]. These changes are more pronounced in hematological malignancies than in solid tumors, leading to impaired function and reduced melatonin secretion.

Malignant diseases can disrupt circadian rhythm regulation via the suprachiasmatic nucleus, impairing pineal gland function. Even in early stages, patients may exhibit altered melatonin levels, contributing to disease progression and complications [7,8]. These disruptions weaken immune responses, increase oxidative stress, and deteriorate overall health.

Chemotherapy further impacts the pineal gland, reducing melatonin secretion with prolonged cycles [9]. This leads to circadian rhythm disturbances, poor sleep, and increased oxidative stress, weakening immune function and potentially worsening disease outcomes.

Despite reduced secretion, melatonin remains a promising adjuvant therapy. Studies suggest its role in inhibiting tumor growth, modulating inflammation, and reducing angiogenesis. It may alleviate chemotherapy side effects, improve sleep, and enhance quality of life [2,4]. Maintaining pineal function could support immune balance and personalized treatment approaches.

In metastatic disease, diminished pineal activity accelerates progression and worsens outcomes. Understanding this relationship is key to identifying biomarkers for monitoring therapy effects and developing strategies to preserve pineal function [5,10].

This study aims to examine pineal gland volume differences between oncology patients and healthy controls, assessing structural changes linked to malignancies. It also investigates the influence of age, sex, and primary tumor origin on pineal volume. Additionally, the study explores the correlation between pineal volume and chemotherapy duration, providing insight into treatment-related changes. To our knowledge, no prior study has evaluated pineal gland volume in oncology patients using MRI in relation to chemotherapy duration. However, given the retrospective design and absence of baseline scans, the interpretation of findings requires caution.

## 2. Materials and Methods

### 2.1. Study Participants

This retrospective study included 400 participants whose MRI scans, obtained between 2014 and 2024, were available in the institutional database. The participants were divided into two groups:

1. Oncology patient group: 200 participants (88 males—44% and 112 females—56%), with an average age of 56.22 ± 9.66 years.
2. Control group: 200 participants (77 males—38.5% and 123 females—61.5%), with an average age of 46.51 ± 15.57 years.

In oncology patients, brain MRI was performed in the context of staging or suspected CNS involvement, according to local oncology practice and clinical guidelines.

### 2.2. Inclusion and Exclusion Criteria

Participants of both sexes were included in the study, provided that a head MRI scan was available in the database of the Oncology Institute of Vojvodina.

For oncology patients, the following exclusion criteria were applied: age below 18 years, presence of a pineal gland cyst, central nervous system infections, vascular malformations, active substance abuse, contraindications for MRI, history of traumatic brain injury, psychiatric disorders (e.g., depression, schizophrenia), dementia/Alzheimer’s disease, multiple sclerosis, primary CNS tumors, and current melatonin supplementation.

For healthy controls, the exclusion criteria were: age below 18 years, presence of a pineal gland cyst, focal or diffuse lesions in white or gray matter (tumors, metastases, congenital anomalies), active opportunistic CNS infections, vascular malformations, history of brain irradiation, active substance abuse, and contraindications for MRI.

The study was approved by the institutional ethics committee, and due to the retrospective nature of the study, informed consent was not required.

### 2.3. Patient Data

Data collected from the information system of the Oncology Institute of Vojvodina included: primary tumor origin and duration of chemotherapy expressed in months. Information on chemotherapy duration was available for 67/200 patients (33.5%), while data on primary tumor origin was available for 112/200 patients. Missing data were primarily due to the fact that a considerable proportion of patients were referred from other hospitals without complete medical documentation accessible in our institutional database. For the subgroup analysis according to tumor origin, only primary tumors with a frequency greater than five across the entire cohort were considered (breast cancer, lung cancer, and melanoma).

### 2.4. Imaging Analysis

MRI examinations were acquired using two clinical scanners: a 1.5 T system (Siemens Aera, Erlangen, Germany) and a 3 T system (Siemens Trio Tim, Erlangen, Germany). Due to the retrospective nature of the study, scan protocols were not entirely uniform. All subjects underwent conventional MRI, which included sagittal T1W, axial T2W, axial FLAIR, axial DWI, and coronal T2W sequences. In addition, oncology patients underwent axial T1W sequences prior to intravenous contrast administration, followed by axial T1W and sagittal 3D T1W MPRAGE sequences after contrast injection.

**Figure 1:**
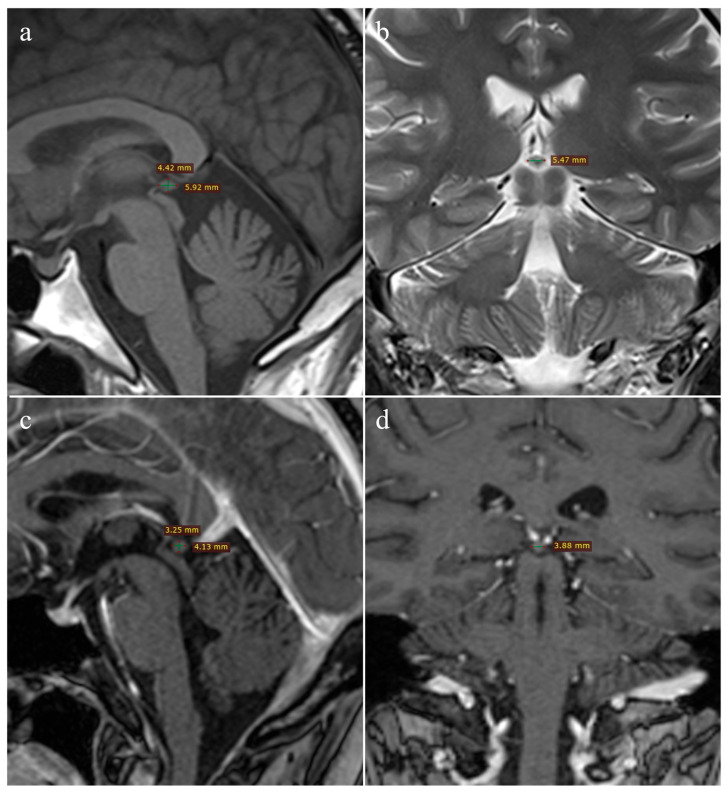
Pineal gland measurements: healthy control (**a**,**b**)—(**a**) sagittal T1W and (**b**) coronal T2W images; oncology patient (**c**,**d**)—3D T1W MPRAGE images in (**c**) sagittal and (**d**) coronal planes.

All image analyses were performed on a Leonardo workstation by two independent readers, who reached decisions in consensus. The pineal gland’s maximum length (L) and height (H) were measured on sagittal T1W images, whereas its width (W) was assessed on coronal or axial T2W images, following the methodology described by Sumida et al. [11]. In oncology patients, measurements of the pineal gland diameters were obtained from post-contrast 3D T1W MPRAGE images with a slice thickness of 1 mm. The gland volume was calculated as an ellipse approximation, using the formula V = (L × H × W)/2 (Figure 1).

To evaluate measurement reliability, pineal gland volume was measured twice in all oncology patients (*n* = 200) by the same reader (intra-rater) and independently by a second reader (inter-rater). Intraclass Correlation Coefficients (ICC) were calculated using a two-way mixed-effects model with absolute agreement.

### 2.5. Statistical Analysis

Statistical analyses were conducted using SPSS software, version 27.0 (IBM Corp., Armonk, NY, USA). A 95% confidence interval was applied, with statistical significance defined as *p* < 0.05.

Group comparability was tested using chi-square test for sex distribution and Mann–Whitney U test for age. Group differences in pineal gland volume and age, stratified by sex, were tested using the Mann–Whitney U test, as normality (Kolmogorov–Smirnov test) was not met. The same test was applied to assess sex-related differences within both groups. Statistical analysis of pineal gland volume among patients with different primary tumors (breast cancer, lung cancer, and melanoma) was conducted using the Kruskal–Wallis test. Spearman’s rank correlation coefficient (ρ) was applied to assess the association between age and pineal gland volume in both groups and sexes, in order to control for disease-related effects. Additionally, an analysis of covariance (ANCOVA) with age as a covariate was performed to assess whether the differences in pineal gland volume between oncology patients and controls remained significant after adjusting for age. Statistical analysis of the effect of chemotherapy duration on pineal gland volume was performed using Spearman’s correlation coefficient. We also compared demographic and volumetric characteristics between patients with and without available chemotherapy data to assess potential systematic differences.

## 3. Results

### 3.1. Demographic Data

A total of 400 participants were included in the study, comprising 200 oncology patients and 200 healthy controls. The oncology group consisted of 88 males (44%) and 112 females (56%), with a mean age of 56.22 ± 9.66 years (range 22–75 years). The control group included 77 males (38.5%) and 123 females (61.5%), with a mean age of 46.51 ± 15.57 years (range 19–82 years). Demographic data are detailed in Table 1, which provides an overview of the distribution of participants by age and sex. There was no significant difference in sex distribution between oncology patients (56.0% female, 44.0% male) and controls (61.5% female, 38.5% male; χ^2^ = 1.25, *p* = 0.26). Oncology patients were significantly older than controls (Mann–Whitney U = 27,914, z = 6.85, *p* < 0.001).

### 3.2. Differences in Volume Between Oncology Patients and Healthy Controls

**Figure 2:**
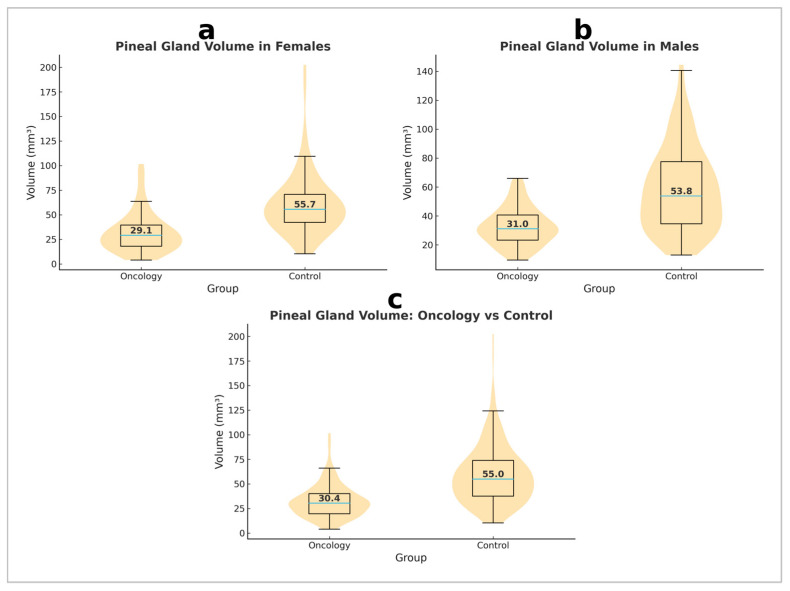
Violin plots with overlaid box plots of pineal gland volume in oncology patients and controls, shown separately for females (**a**), males (**b**), and the entire cohort (**c**).

Analysis of pineal gland volume demonstrated a significant difference between oncology patients and healthy controls (*p* < 0.001, Mann–Whitney U test) (Figure 2). An ANCOVA with age as a covariate confirmed that pineal gland volume remained significantly smaller in oncology patients compared to controls (F(1, 397) = 114.584, *p* < 0.001, partial η^2^ = 0.224), while age itself did not significantly influence pineal volume (F(1, 397) = 1.063, *p* = 0.303). These results indicate a significantly reduced pineal gland volume in oncology patients compared to healthy controls, both in the entire sample and by sex (Table 2), suggesting the presence of pathological mechanisms affecting the function or structure of the pineal gland in oncology patients.

Reliability analysis demonstrated excellent reproducibility. Intra-rater reliability showed an ICC of 0.988 (95% CI: 0.984–0.991, *p* < 0.001, Single Measures) and 0.994 (95% CI: 0.992–0.995, *p* < 0.001, Average Measures). Inter-rater reliability was also excellent, with an ICC of 0.982 (95% CI: 0.977–0.986, *p* < 0.001, Single Measures) and 0.991 (95% CI: 0.988–0.993, *p* < 0.001, Average Measures). These findings confirm the robustness and reproducibility of pineal gland volume estimation.

### 3.3. Sex Differences in Pineal Gland Volume Within the Control Group

The mean pineal gland volume was 58.35 ± 28.70 mm^3^ in healthy females and 60.72 ± 32.09 mm^3^ in healthy males. No statistically significant sex-related difference was observed in the control group (*p* = 0.683). These results suggest that sex does not significantly influence pineal gland volume in the healthy population.

### 3.4. Sex Differences in Pineal Gland Volume Within the Oncology Group

The mean pineal gland volume was 32.77 ± 13.00 mm^3^ in female patients and 31.96 ± 20.71 mm^3^ in male patients. Analysis of differences between sexes in the oncology group did not reveal a statistically significant difference (*p* = 0.110). These results indicate that, despite the significantly reduced pineal gland volume in oncology patients compared to healthy controls, sex was not a factor significantly contributing to variations in volume within the oncology group.

### 3.5. Differences in Pineal Gland Volume Depending on Primary Tumor Origin

**Figure 3:**
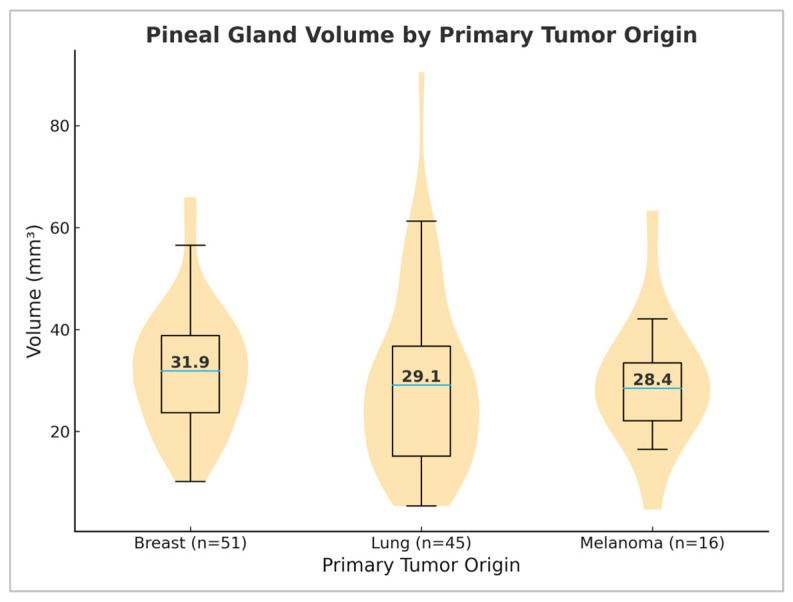
Violin plots with overlaid box plots of pineal gland volume by primary tumor origin, including patients with breast cancer (*n* = 51), lung cancer (*n* = 45), and melanoma (*n* = 16). Median values are indicated within each distribution.

The analysis of tumor origin was possible in 112/200 patients and was limited to primary tumors with a frequency greater than five. The subgroup included patients with breast cancer (*n* = 51), lung cancer (*n* = 45), and melanoma (*n* = 16) as the most common tumors in the oncology group. Given the reduced sample size, these results should be interpreted as underpowered and preliminary. The average pineal gland volume was 32.03 ± 12.09 mm^3^ in patients with breast cancer, 29.57 ± 17.40 mm^3^ in patients with lung cancer, and 29.51 ± 12.74 mm^3^ in patients with melanoma. Statistical analysis using the Kruskal–Wallis test did not indicate significant differences in pineal gland volume between the groups (*p* = 0.296, *p* > 0.05) (Figure 3). These findings suggest that primary tumor origin does not significantly affect pineal gland volume.

### 3.6. Correlation Between Age and Pineal Gland Volume

No significant correlation was found between age and pineal gland volume in healthy controls (ρ = 0.08; *p* > 0.05) or oncology patients (ρ = −0.087; *p* > 0.05). Analysis of the entire cohort likewise showed no correlation (ρ = 0.052; *p* = 0.303) (Table 3). These findings suggest that age does not significantly influence pineal gland volume, regardless of group affiliation. Such results support the hypothesis that other physiological and pathological characteristics, such as disease or other risk factors, are likely more responsible for variations in pineal gland volume than age itself.

### 3.7. Correlation Between Chemotherapy Duration and Pineal Gland Volume

**Figure 4:**
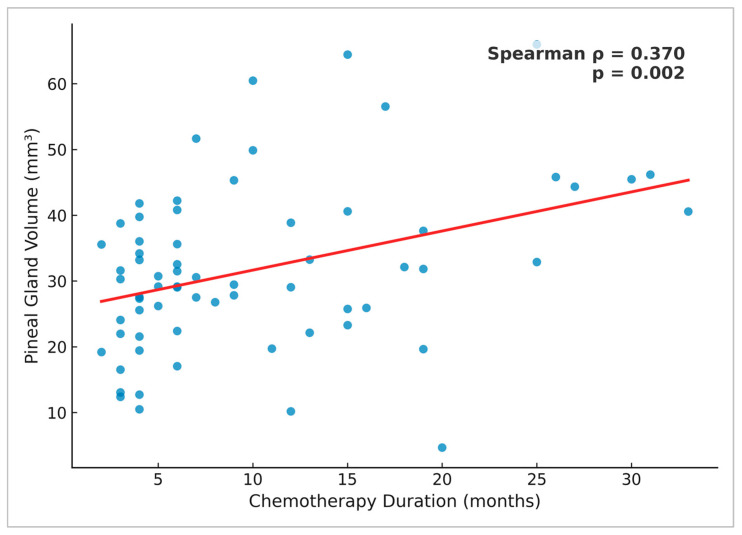
Scatter plot showing the correlation between pineal gland volume and chemotherapy duration (*n* = 67). Each point represents an individual patient. The red line indicates the linear regression fit. Spearman’s analysis demonstrated a moderate positive correlation (ρ = 0.370, *p* = 0.002).

In the subgroup of 67 oncology patients with available chemotherapy duration data, Spearman’s analysis demonstrated a moderate positive correlation between pineal gland volume and chemotherapy duration (ρ = 0.370, *p* = 0.002) (Figure 4). To assess potential systematic differences, we compared patients with available chemotherapy duration data (*n* = 67) and those without (*n* = 133). There was no significant difference in pineal gland volume between the two groups (Mann–Whitney U = 4617.5, z = 0.42, *p* = 0.68). However, sex distribution differed significantly, with women being more frequently represented among patients with available chemotherapy data (χ^2^ = 19.10, *p* < 0.001).

## 4. Discussion

To the best of our knowledge, this is the first study that has examined pineal gland volume in oncology patients using a simple methodological approach, while simultaneously investigating the correlation between pineal gland volume and the duration of chemotherapy. The exact causes of pineal gland atrophy are not fully understood. For this reason, this neuroendocrine structure has been the subject of numerous studies for decades, aiming to explain its mechanisms of action and facilitate therapeutic approaches for oncology patients.

Analysis of pineal gland volume between the groups revealed significantly reduced pineal gland volume in oncology patients compared to healthy controls, even after adjusting for age. The interpretation of this finding remains complex, and three non-exclusive explanations should be considered. First, it is possible that a smaller pineal gland volume represents a pre-existing condition, independent of malignancy or its treatment. Second, reduced volume may reflect disease-related factors, such as systemic effects of cancer, tumor burden, or cancer-associated inflammation. Third, chemotherapy or other oncological treatments may directly or indirectly contribute to pineal gland alterations. Given the retrospective design and absence of pre-treatment MRI scans, our study cannot distinguish between these scenarios, and no single explanation should be given undue weight. Future prospective studies with baseline imaging are required to clarify these relationships.

Numerous studies have demonstrated a reduction in pineal gland volume depending on the presence of various diseases such as schizophrenia [12], Alzheimer’s disease [13], mood disorder [14], depression [15] and sleep disorders [16], indicating the influence of different pathophysiological mechanisms on pineal gland morphology. In addition to the mentioned disorders, Batin et al. in their study also confirmed the decrease in the pineal gland volume in the development of adolescent idiopathic scoliosis (AIS) [17]. Hajdu et al. described irreversible morphological changes in the pineal gland in autopsies of 275 oncology patients, including calcification, cystic cavities, and gliosis. Their study, which included pediatric patients, describes similar changes as in adults, suggesting the influence of tumor processes or therapeutic interventions [4,18].

In our study, no difference between sexes was found, either in healthy controls or in oncology patients, suggesting that sex is not a significant factor influencing pineal gland volume. Additionally, no correlation was found between age and pineal gland volume in either oncology patients or the control group. Although the oncology group was significantly older than the controls, while sex distribution was identical between groups, ANCOVA demonstrated that age did not significantly influence pineal gland volume and that group differences remained highly significant. These results are consistent with other studies [11,19,20,21]. Some studies have observed a correlation between age and volume [22], which may be due to different methodologies. Our approach to measuring volume corresponds to the methodology of Sumida et al., who calculated volume as an ellipse approximation using the formula V = (L × H × W)/2 (Figure 1) [11]. Acer et al., comparing three methods for measuring pineal gland volume using MRI, obtained results consistent with ours, despite different methodologies [20]. Although volume estimation was performed using an elliptical approximation on 2D slices, the very high intra- and inter-rater ICC values (>0.98) demonstrate excellent reproducibility and support the validity of our measurement approach.

In examining the difference in pineal gland volume depending on primary tumor origin, no significant difference was found between subgroups of patients with breast cancer, lung cancer, and melanoma. Previous studies, using different methodologies, have observed melatonin levels in different primary tumors. Dogliotti et al. [23] found elevated serum melatonin levels in patients with locally advanced tumors and metastases, while the results of Tamarkin et al. showed the opposite [24]. A potential explanation lies in the fact that melatonin levels decrease with the growth of the primary tumor while it is localized, and then increase when distant metastases appear [10].

To date, no study has examined changes in pineal gland volume in relation to the chemotherapy duration. The observed moderate positive correlation between chemotherapy duration and pineal gland volume should be interpreted with caution. A plausible explanation is survivorship bias, as patients surviving longer and tolerating extended chemotherapy may constitute a subgroup with better-preserved pineal morphology, rather than reflecting a direct therapeutic effect. Lissoni et al. found significantly lower melatonin concentrations in patients undergoing chemotherapy, but it is not fully understood whether the drop in melatonin levels represents an adverse effect of treatment. The reduction in melatonin levels may be due to direct damage to the pineal gland by chemotherapeutic agents, given the absence of the blood–brain barrier at the level of the gland [5,25].

Numerous experimental studies have highlighted the role of melatonin in cancer treatment and prevention. Talib et al. described the anticancer effects of melatonin against malignancies of various origins, including colorectal cancer, breast cancer, lung cancer, prostate cancer, and gastric cancer. This effect is mediated by mechanisms such as immunomodulatory action, inhibition of angiogenesis, induction of apoptosis, and antimetastatic effects. The combination of melatonin and chemotherapy has shown improved efficacy of conventional therapy and quality of life in patients [1,25,26,27].

Our study has several limitations. One of them is the small sample size of certain subgroups, particularly patients with available data on chemotherapy duration (67/200 participants) and primary tumor origin (112/200 participants). These data were incomplete, largely due to missing documentation in patients referred from other hospitals. Although patients with and without chemotherapy duration data did not differ significantly in terms of age, sex, or pineal volume, this limitation may still have introduced bias. In addition, the tumor origin analysis was based on only three cancer types, making these results underpowered and preliminary, and limiting the generalizability of the findings. This study was retrospective and lacked pre-chemotherapy MRI scans, preventing longitudinal assessment. Data on chemotherapy regimens, TNM staging, and reproductive cycle in women were not consistently available, as many patients were referred from other hospitals. Serum melatonin levels and sleep quality assessments were not included, limiting functional interpretation of pineal volume findings. Addressing this potential limitation would further enhance the significance of the research, considering that study by Hinz et al. confirmed severe sleep problems in oncology patients by examining the Pittsburgh Sleep Quality Index (PSQI) as the most frequently used questionnaire for assessing sleep quality [28].

Despite these limitations, our study provides the first volumetric MRI assessment of the pineal gland in oncology patients and highlights the need for prospective studies with baseline imaging and biochemical markers to better understand pineal gland changes in cancer.

## 5. Conclusions

This study highlights a significantly reduced pineal gland volume in oncology patients compared to controls, independent of sex and age. No significant differences were observed based on primary tumor origin. A moderate positive correlation was found between chemotherapy duration and pineal gland volume; however, this association should be interpreted cautiously, as survivorship bias is a more plausible explanation than direct treatment effects. Further prospective studies with pre-treatment imaging and comprehensive clinical data are required to clarify the mechanisms underlying these findings and their potential clinical implications.

## Figures and Tables

**Table 1:** Demographic characteristics of oncology patients and healthy controls.

Group	*n* (%)	Age ( $\bar{X}$ ± SD)
Oncology patients	200 (100%)	56.22 ± 9.66
Males	88 (44%)	57.22 ± 9.45
Females	112 (56%)	55.45 ± 9.79
Healthy controls	200 (100%)	46.51 ± 15.57
Males	77 (38.5%)	48.51 ± 16.21
Females	123 (61.5%)	45.27 ± 15.09

**Table 2:** Sex-specific differences in pineal gland volume (mm^3^) by group.

Sex	Oncology Patients	Healthy Controls	*p*-Value
Males	31.96 ± 20.71	60.72 ± 32.09	<0.001
Females	32.77 ± 13.00	58.35 ± 28.70	<0.001
Total	32.41 ± 16.79	59.26 ± 29.99	<0.001

**Table 3:** Correlation between age and pineal gland volume by group.

Group	Correlation Coefficient (ρ/r *)	*p*-Value
Oncology patients	−0.087	0.220
Males	−0.044	0.683
Females	−0.163	0.086
Healthy controls	0.080	0.259
Males	0.039	0.734
Females	0.152 *	0.093

* Pearson correlation coefficient.

## Data Availability

The human data supporting the findings of this study are not openly available due to privacy and sensitivity concerns. They are available from the corresponding author upon request.

## References

[1] Talib W.H., Alsayed A.R., Abuawad A., Daoud S., Mahmod A.I. (2021). Melatonin in cancer treatment: Current knowledge and future opportunities. Molecules.

[2] Montaruli A., Castelli L., Mulè A., Scurati R., Esposito F., Galasso L., Roveda E. (2021). Biological rhythm and chronotype: New perspectives in health. Biomolecules.

[3] Bartsch C., Bartsch H., Blask D.E. (2001). The Pineal Gland and Cancer: Neuroimmunoendocrine Mechanisms in Malignancy.

[4] Hajdu S.I., Porro R.S., Lieberman P.H., Foote F.W. (1972). Degeneration of the pineal gland of patients with cancer. Cancer.

[5] Lissoni P., Viviani S., Bajetta E., Buzzoni R., Barreca A., Mauri R., Resentini M., Morabito F., Esposti D., Esposti G. (1986). A clinical study of the pineal gland activity in oncologic patients. Cancer.

[6] Feuer G.M., Kerenyi N.A. (1989). Role of the pineal gland in the development of malignant melanoma. Neurochem. Int..

[7] Desai K., Pereira K., Ali H., Thirumaran R. (2024). Pineal gland: Sleep, malignancy, and statistics. J. Clin. Oncol..

[8] Li Y., Li S., Zhou Y., Meng X., Zhang J.J., Xu D.P., Li H.-B. (2017). Melatonin for the prevention and treatment of cancer. Oncotarget.

[9] Bartsch H., Bartsch C., Mecke D. (2001). Analysis of melatonin in patients with cancer of the reproductive system. The Pineal Gland and Cancer.

[10] Lapin V., Bartsch C., Bartsch H., Blask D.E., Bartsch C., Bartsch H., Blask D.E. (2001). Pineal gland and malignancy. Neuroimmunoendocrine Mechanisms in Malignancy.

[11] Sumida M., Barkovich A.J., Newton T.H. (1996). Development of the pineal gland: Measurement with MR. Am. J. Neuroradiol..

[12] Takahashi T., Nakamura M., Sasabayashi D., Nishikawa Y., Takayanagi Y., Nishiyama S., Higuchi Y., Furuichi A., Kido M., Noguchi K. (2019). Reduced pineal gland volume across the stages of schizophrenia. Schizophr. Res..

[13] Matsuoka T., Oya N., Yokota H., Akazawa K., Yamada K., Narumoto J., Alzheimer’s Disease Neuroimaging Initiative (2020). Pineal volume reduction in patients with mild cognitive impairment who converted to Alzheimer’s disease. Psychiatry Clin. Neurosci..

[14] Chauhan S., Barbanta A., Ettinger U., Kumari V. (2023). Pineal abnormalities in psychosis and mood disorders: A systematic review. Brain Sci..

[15] Gürbüz A.A., Altun H., Tahiroğlu A.Y., Mert G.G., Kızıldağ B., Arslan S.C. (2024). Pineal gland volume in children with intellectual disability. Int. J. Dev. Neurosci..

[16] Park J., Han J.W., Suh S.W., Byun S., Han J.H., Bae J.B., Kim J.H., Kim K.W. (2020). Pineal gland volume is associated with prevalent and incident isolated rapid eye movement sleep behavior disorder. Aging.

[17] Batın S., Ekinci Y., Gürbüz K., Payas A., Kurtoğlu E., Uçar İ., Seber T., Arık M., Yılmaz H., Unur E. (2023). The role of pineal gland volume in the development of scoliosis. Eur. Spine J..

[18] Scharenberg K., Liss L. (1965). The histologic structure of the human pineal body. Prog. Brain Res..

[19] Vuković M., Nosek I., Boban J., Kozić D. (2024). Pineal gland volume loss in females with multiple sclerosis. Front. Neuroanat..

[20] Acer N., Ilıca A.T., Turgut A.T., Özçelik Ö., Yıldırım B., Turgut M. (2012). Comparison of three methods for the estimation of pineal gland volume using magnetic resonance imaging. Sci. World J..

[21] Schmitz S.A., Platzek I., Kunz D., Mahlberg R., Wolf K.J., Heidenreich J.O. (2006). Computed tomography of the human pineal gland for study of the sleep–wake rhythm: Reproducibility of a semi-quantitative approach. Acta Radiol..

[22] Sun B., Wang D., Tang Y., Fan L., Lin X., Yu T., Qi H., Li Z., Liu S. (2009). The pineal volume: A three-dimensional volumetric study in healthy young adults using 3.0 T MR data. Int. J. Dev. Neurosci..

[23] Dogliotti L., Berruti A., Buniva T., Torta M., Bottini A., Tampellini M., Terzolo M., Faggiuolo R., Angeli A. (1990). Melatonin and human cancer. J. Steroid Biochem. Mol. Biol..

[24] Tamarkin L., Cohen M., Roselle D., Reichert C., Lippman M., Chabner B. (1982). Decreased nocturnal plasma melatonin peak in patients with estrogen receptor positive breast cancer. Science.

[25] Ziegler I., Maier K., Fink M. (1982). Pteridine-binding I-acid glycoprotein from blood of patients with neoplastic diseases. Cancer Res..

[26] Pourhanifeh M.H., Mahdavinia M., Reiter R.J., Asemi Z. (2019). Potential use of melatonin in skin cancer treatment: A review of current biological evidence. J. Cell. Physiol..

[27] Luo J., Zhang Z., Sun H., Song J., Chen X., Huang J., Lin X., Zhou R. (2020). Effect of melatonin on T/B cell activation and immune regulation in pinealectomy mice. Life Sci..

[28] Hinz A., Friedrich M., Schulte T., Petrowski K., Tibubos A.N., Hartung T.J. (2025). The Pittsburgh Sleep Quality Index (PSQI) applied to cancer patients: Psychometric properties and factors affecting sleep quality. Cancer Investig..

